# Aggravating effects of treadmill exercises during the early-onset period in a rat traumatic brain injury model: When should rehabilitation exercises be initiated?

**DOI:** 10.1016/j.ibror.2019.10.002

**Published:** 2019-10-22

**Authors:** Satoru Taguchi, Mohammed E. Choudhury, Kazuya Miyanishi, Yuiko Nakanishi, Kenji Kameda, Naoki Abe, Hajime Yano, Toshihiro Yorozuya, Junya Tanaka

**Affiliations:** a Department of Molecular and Cellular Physiology, Graduate School of Medicine, Ehime University, Toon, Ehime, Japan; b Advanced Research Support Center, Division of Analytical Bio-Medicine, Ehime University, Toon, Ehime, Japan; c Department of Anesthesia and Perioperative Medicine, Graduate School of Medicine, Ehime University, Toon, Ehime, Japan

**Keywords:** Cylinder test, Beam test, IL-1β, CCL2, TGF β, IL-6

## Abstract

Physical exercise is one of the best interventions for improving traumatic brain injury (TBI) outcomes. However, an argument has been raised regarding the timing at which physical exercise should be initiated. In this study, male Wistar rats were subjected to stab wounding of the right hemisphere to develop a TBI model and were forced to walk once on a treadmill at a 5-m/min pace at 24 h or 48 h after TBI for 10 min. Injured brain tissue was dissected after TBI to evaluate the effects of exercise. Behavioral abnormalities and motor impairment were assessed by various behavioral tests between 2 and 3 weeks after TBI. Exercise did not affect the circulating corticosterone levels and the weight of the adrenal glands. Exercise particularly that at 24 h, worsened the motor impairment of the left forelimbs. Quantitative reverse-transcription polymerase chain reaction showed that exercise at 24 h increased proinflammatory cytokines and chemokines on the third day while suppressing the proinflammatory reactions on the fourth day. Exercise at both time points decreased expression of transforming growth factor (TGF) β1 and its receptor TGFβR1. Exercise at 24 h increased phosphorylation of IκB kinase on the fourth day, which may be correlated with the decreased effects of TGFβ1. Even a low-intensity exercise activity could cause deleterious effects when it is initiated within 48 h after the onset of severe TBI, probably because of the resulting proinflammatory effects. Therefore, rehabilitation exercise programs should be initiated after 48 h of TBI onset.

## Highlights

• A traumatic brain injury model was prepared in rats by stab wounding.
• Rats were forced to walk slowly on a treadmill once for 10 min at 24 h or 48 h after wounding.
• Exercise, particularly at 24 h, aggravated motor impairment while increasing the expression of proinflammatory factors.
• Exercise for rehabilitation should be initiated after 48 h of severe brain injury onset.

## Graphical abstract

**Figure:**
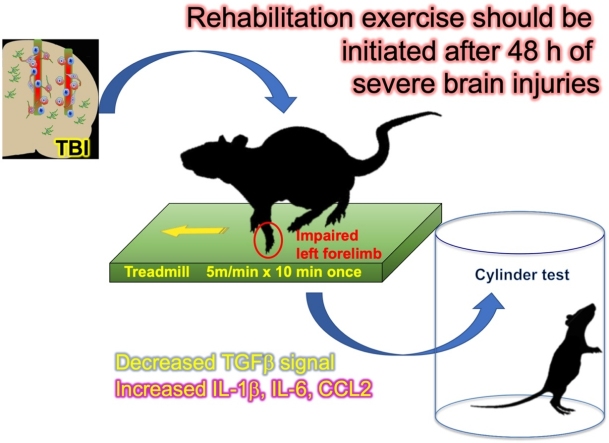

## 1 Introduction

Traumatic brain injury (TBI) causes more than one-third of all injury-related deaths (Majdan et al., 2016). Moreover, TBI is one of the leading causes of incurable neurological disability and/or psychological problems affecting several million people worldwide every year (Corps et al., 2015; Gyoneva and Ransohoff, 2015). The sequelae following TBI include personality changes, deficits in learning and memory, an increased risk of neurodegenerative changes, such as Alzheimer’s disease in addition to chronic traumatic encephalopathy (Griesbach, 2011; McKee et al., 2013). The incidence of TBI has increased in recent years, partly due to the growth of the elderly population (Ramanathan et al., 2012). Despite the serious pathological conditions and intensive research, established interventions to ameliorate the outcome of TBI are limited. Among a few options, rehabilitation exercise programs may exert the best ameliorative effects (Griesbach, 2011; Rimmer et al., 2010).

Ameliorative effects of physical exercises as rehabilitation have been extensively investigated for brain injuries such as TBI and stroke, both in clinical and laboratory settings. However, mechanisms underlying the benefits at the molecular and cellular levels are still to be elucidated. One of the frequent arguments that arise regarding rehabilitation research is the timing of initiation for severe brain injuries. Many studies have shown that initiating exercise early after the TBI onset, such as between 24–72 h after the event onset, is more beneficial than a later initiation (Griesbach, 2011; Maulden et al., 2005; Shen et al., 2016; Tong et al., 2019; Yoon and Kim, 2018). Initiation, even at very early time points or within 24 h, has been recommended (Bernhardt et al., 2008; Tian et al., 2013; Zhang et al., 2012). Yet, aggravating effects of physical exercise during early or very early periods after the severe TBI have also been demonstrated (Li et al., 2017a, b; Risedal et al., 1999; Shen et al., 2016). Many studies have attributed the aggravating effects of physical exercise during the early period to enhanced proinflammatory reactions in and around the lesion that can lead to further neural cell death.

However, many of these studies, addressing the timing to start rehabilitation exercise, employed prolonged periods of exercise for days and weeks, thereby the studies did not analyze solely the effect of the timing of initiation. This study was aimed to determine which is the better timing 24 h or 48 h after TBI to initiate exercise as rehabilitation. For this aim, we employed one-time treadmill walking just for 10 min at a speed 5 m/min at 24 h or 48 h after TBI and analyzed the outcome using various behavioral tests and also investigated the effects of the exercise on molecular and cellular responses in the injured tissues.

## 2 Materials and methods

### 2.1 Animals

All animal experiments were carried out in accordance with the Guidelines for Animal Experimentation of Ehime University Graduate School of Medicine. Male Wistar rats (8- to 9-weeks-old, with a body weight of 270–300 g) were housed under standard laboratory conditions, where light on was set at 7:00 and light off at 19:00.

### 2.2 Preparation of the TBI model

TBI was achieved by a stab wound, mainly targeting the cerebral cortex and the striatum (Yokoyama et al., 2006). Rats were anesthetized with isoflurane, and a 15-mm longitudinal incision was made in the skin to expose the skull. Two holes were made through the skull over the right hemisphere at approximately 2.5 and 4 mm to the right of the midline and 1 mm posterior to the bregma. Through each hole, a 26-gage needle was inserted to a depth of approximately 7 mm from the surface of the skull, and the needle was moved in an anteroposterior direction parallel to the midline. Thereafter, the needle was withdrawn, and the skin incision was closed with quick-drying glue (Aron-Alpha; Toagosei, Tokyo, Japan). Sham operation was done all the procedure described above other than the insertion of the needle into the brain parenchyma.

### 2.3 Experimental design and treadmill exercise

**Fig. 1:**
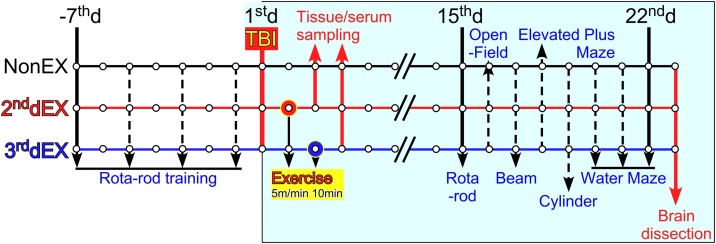
Experimental schedule for the pre- and post-TBI periods. Pretraining for the rotarod test was done four times for 1 week before wounding. Rats with TBI were divided into three groups: non-exercise (NonEX), exercise on the 2^nd^ day (2^nd^dEX), or exercise on the 3^rd^ day (3^rd^dEX). The treadmill exercise was done at 5 m/min for 10 min. Two weeks after the forced exercise, behavioral tests (rotarod, open-field, beam, elevated plus maze, cylinder, and water maze) were performed for 8 days.

The experimental design is shown in Fig. 1. Rats were assigned to three groups: non-exercise (NonEX), exercise on the second day (or 24 h after TBI; 2^nd^dEX), and exercise on the third day (or 48 h after TBI; 3^rd^dEX). NonEx rats were kept in the normal cages. Rats assigned to the exercise groups were forced to walk on a treadmill once for 10 min. When the rats stopped walking, they received an electrical shock (75 V alternating current) with a shock grid at the end of the belt.

### 2.4 Behavioral tests

Various behavioral tests (rotarod, open-field, beam, elevated plus maze, cylinder, and water maze) were done from 19:00 by 22:00 (Miyanishi et al., 2019; Abe et al., 2018; Pietrelli et al., 2012).

#### 2.4.1 Rota-rod test

The rota-rod test was done to evaluate motor coordination and balance using a rota-rod apparatus (Ugo Basile, Rota-rod 7750, Italy). The speed of the rota-rod was accelerated from 4 to 20 rpm in 1 min.

#### 2.4.2 Open-field test

The open-field test was done to evaluate activity, anxiety and depressive mood of rats using a square open field (100 × 100 cm) with 50 cm high walls, equipped with a video-tracking system (Ethovision XT 7, Noldus Info. Tech., Wageningen, The Netherlands). The rats were placed in a corner of the open-field. The movement was video-recorded for 5 min. The total distance moved, the latency to the first entrance into and the duration in the center zone (50 × 50 cm) set in the middle of the field were measured.

#### 2.4.3 Beam test

The beam test was done to assess motor coordination and balance. The rats walked on a wooden beam suspended between a start platform and their home cage at a height of 50 cm. The rats were put on the start platform and the foot slips were evaluated by a 7-category rating system using a scale of 0–6 as described elsewhere (Miyanishi et al., 2019).

#### 2.4.4 Cylinder test

The cylinder test was conducted to evaluate the motor deficits in the left forelimb that assesses voluntary movement in a transparent glass cylinder. An experimenter counted the number of wall contacts with each forelimb in 5 min.

#### 2.4.5 Morris water maze test

Spatial memory was assessed using a standard Morris water maze test using a 150-cm diameter × 45-cm tall circular pool filled with water at 25 °C. A 12-cm diameter circular transparent platform was placed 2 cm below the waterline in the center of one quadrant. The number of rats accomplishing the task was recorded and the mean latency to the platform, total distance swum, and the swim speed was measured for each trial using the video-tracking system. In the probe test, duration in the quadrant where the platform was set in the previous tests was also recorded. The trials were done for three sequential days.

#### 2.4.6 Elevated plus maze test

Elevated plus maze test was done to evaluate activity and anxious behavior. Elevated plus maze made of black plastic was set at 50 cm high, with cross shaped open and closed arms, 50 cm long and 12 cm wide.

### 2.5 Evaluation of TBI-induced brain tissue volume loss

Brains of TBI rats were dissected on the 23^rd^ day and incubated for 7 d in a 4% paraformaldehyde solution made with phosphate buffered saline (PBS). The fixed brains were coronally sliced with a 2-mm thickness (Abe et al., 2018). The seven slices were photographed, and areas of lost tissue in the right hemisphere were measured using Photoshop® and ImageJ 1.50i. The severity of brain tissue loss was evaluated by dividing the areas of tissue loss by the total area of the left hemisphere.

### 2.6 RNA isolation from brains and quantitative reverse-transcription-polymerase chain reaction (qPCR)

On the 3^rd^ and 4^th^ d, TBI rats underwent deep anesthesia, and their brains were dissected. Total RNA was extracted from homogenates of injured tissue around the stab wounds using an RNeasy Lipid Tissue Mini Kit (Qiagen, Hilden, Germany) as previously described (Abe et al., 2018). All gene-specific mRNA measurements were normalized to glyceraldehyde 3-phosphate dehydrogenase (GAPDH) mRNA levels. All PCR primer sequences are listed in Supplementary Table 1.

### 2.7 Fluorescence-activated cell sorting and qPCR

Under deep anesthesia, TBI rat brains were dissected on the 3^rd^ and 4^th^ d after perfusion with chilled PBS for 3 min to remove blood. Right brain hemispheres (∼500 mg) were dissociated into single cells in a gentleMACS Dissociator as previously described (Abe et al., 2018). The cell suspensions were diluted to 1 × 10^6^ cells/100 μl. Fc receptors were blocked using a purified mouse anti-Rat CD32 antibody (BD Pharmingen, Franklin Lakes, NJ), and cells were incubated with fluorescence-labeled antibodies listed in Supplementary Table 2. Dead cells were sorted out using Zombie NIR (BioLegend, San Diego, CA). Before flow cytometry sorting, dissociated cells were resuspended with 10 volumes of Cell Cover (AL Anacyte Laboratories UG, Hamburg, Germany) at 4 °C to stabilize mRNA. Macrophages and microglial cells were collected on a BD FACSAria flow cytometer (BD biosciences, Franklin Lakes, NJ). Data were analyzed using FlowJo Software (version.7.6.5, Treestar, Ashland, OR). Total RNA was prepared from cells using an RNeasy micro kit (Qiagen) and then reverse-transcribed to obtain cDNA.

### 2.8 Immunoblotting

TBI rat brains were dissected on the 3^rd^ and 4^th^ d for immunoblotting. Rectangularly dissected contralateral and ipsilateral brain tissues were homogenized with Laemmli’s sample solution containing 3% sodium dodecyl sulfate for immunoblotting as described elsewhere (Sugimoto et al., 2014). The blots were incubated with antibodies listed in Supplementary Table 3. Immunoreactive bands were analyzed by densitometry using ImageJ 1.50i.

### 2.9 Plasma corticosterone levels and adrenal gland weight

On the 3^rd^ and 4^th^ d, heparinized blood of rats was taken by heart puncture, and bilateral adrenal glands were dissected. Plasma corticosterone levels were determined as described elsewhere (Nishioka et al., 2016) using an ELISA kit (Arbor Assays, Ann Arbor, MI, USA). The weight of the dissected adrenal glands was measured.

### 2.10 Statistical analysis

Data expressed as means ± SEM or SD were statistically analyzed using InStat3 software (GraphPad Software, La Jolla, CA, USA). Data were subjected to two-tailed Student’s *t*-test (paired) or one-way ANOVA with Tukey’s post hoc test. Significance was set at P < 0.05.

## 3 Results

### 3.1 Effects of exercise on plasma corticosterone levels and body weight

**Fig. 2:**
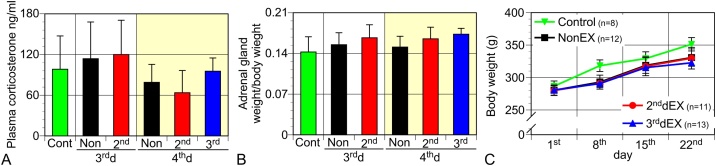
TBI and the one-time short-period exercise did not affect either plasma corticosterone level (A), adrenal gland weights (B) or body weight gain (C). Control (Cont) denotes the data from the normal rats. Non, 2^nd^, and 3^rd^ in the graphs denote, NonEX, 2^nd^dEX, and the 3^rd^dEX group respectively. Plasma and adrenal glands were taken on the 3^rd^ and 4^th^ d. (A) Plasma corticosterone levels were determined by ELISA. (B) The weight of the dissected adrenal glands was measured, which was normalized according to the body weight of the rats. Data in A and B (n = 4 for data on the 4^th^ d; n = 5 for control and those on the 3^rd^ d) are shown as mean ± SEM (A) or SD (B). (C) Body weight changes after TBI. TBI weakly suppressed body weight gain and exercise did not affect body weight. Data are expressed as mean ± SD.

A rat TBI model was developed by means of a stab wound injury in the forebrain of male Wistar rats. Rats were forced to walk on the treadmill (5 m/min) for 10 min once at 24 h or 48 h after TBI. Blood samples were taken on the 3^rd^ d or 4^th^ d or 24 h or 48 h after the forced exercise session to determine the corticosterone levels. On the same days, the adrenal glands were dissected and weighed (Fig. 2). Body weight was measured weekly after TBI to record any changes. Exercise for groups 2^nd^dEX and 3^rd^dEX did not affect the level of corticosterone, body weight, or adrenal gland weight, indicating that the exercise was not very stressful.

### 3.2 Effects of exercise on outcomes of TBI

**Fig. 3:**
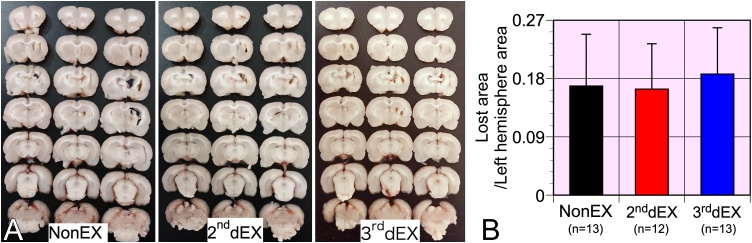
Brain tissue volume loss after TBI. (A) Representative images (n = 3) of sliced TBI brain tissue of rats in each group. (B) Exercise did not affect volume loss of injured brain tissue. Data from 12 or 13 rats in each group are shown as mean ± SD.

**Fig. 4:**
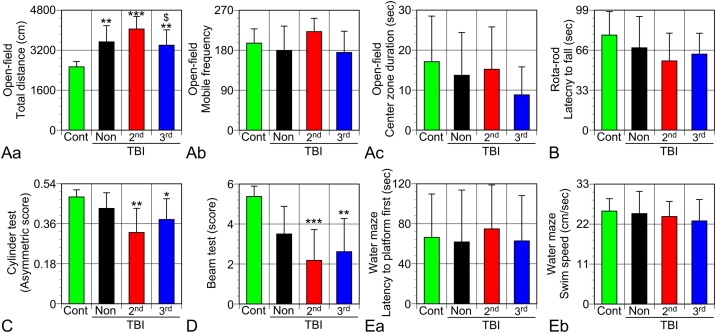
Effects of the one-time exercise session on the behavior of TBI rats. (A) TBI increased the activity of rats as measured by the total distance moved in the open-field test (Aa), whereas mobile frequency (Ab) and duration (data not shown) were not significantly changed. “Cont” denotes the healthy control rats with almost the same body weights as TBI rats. The 2^nd^dEX rats walked the longest among each group. The total distance walked by 3^rd^dEX rats was significantly shorter than that by 2^nd^dEX rats. (Ac) Time spent at the center zone of the open-field test was not significantly different among the groups. (B) No significant changes were found in the rotarod test. (C) The cylinder test detected significant exercise-induced aggravation in the asymmetric score, an indicator of motor deficit of the left forelimbs. (D) The beam test also showed significant aggravation of motor dysfunction in the EX groups. (E) TBI did not affect the spatial cognitive functions as well as swim speed as revealed by the Morris water maze test. Data (Cont n = 7, NonEX and 2^nd^dEX n = 10, 3^rd^dEX n = 11) are shown as mean ± SD. *, **, *** indicate P < 0.05, 0.01, 0.001, respectively vs. Cont. $ indicates P < 0.05, vs. 2^nd^dEX.

Exercise did not lead to volume loss of injured brain tissue (Fig. 3A and B). However, behavioral tests from 2 to 3 weeks after TBI detected the aggravating effects of the one-time exercise session (Fig. 4). TBI-caused hyperactivity of rats, as shown in the open-field test (Fig. 4A). TBI rats that exercised on the 2^nd^ d moved the longest distance for 5 min on the open-field. Anxious behavior, evaluated by staying in the center zone of the arena, was not different among the groups. Similarly, exercise did not affect the behavior on the elevated plus maze (data not shown) that is suitable for evaluation of anxious behavior. There were no significant differences in the ability to move on the rotating rod (Fig. 4B). The cylinder test, which can detect hemiplegia of the forelimbs (Miyanishi et al., 2019), showed that the rats both in groups 2^nd^dEX and 3^rd^dEX presented significantly worsened motor deficits of the left forelimb (Fig. 4C). Rats in particular in group 2^nd^dEX had worse results than those in NonEX. The beam test, suitable for detecting deficits in motor coordination and balance, showed aggravating effects of exercise, particularly in group 2^nd^dEX (Fig. 4D). The stab wounding applied to induce the TBI model did not cause cognitive dysfunction as evaluated by the Morris water maze test that showed no significant differences in the number of rats accomplishing the task (not shown), the mean latency to the platform (Fig. 4Ea), total distance swum (not shown), and the swim speed (Fig. 4Eb) was measured for each trial using the video-tracking system. Exercise did not produce any significant results in the probe test in the water maze test (not shown).

### 3.3 Changes in mRNA expression after TBI and exercise

**Fig. 5:**
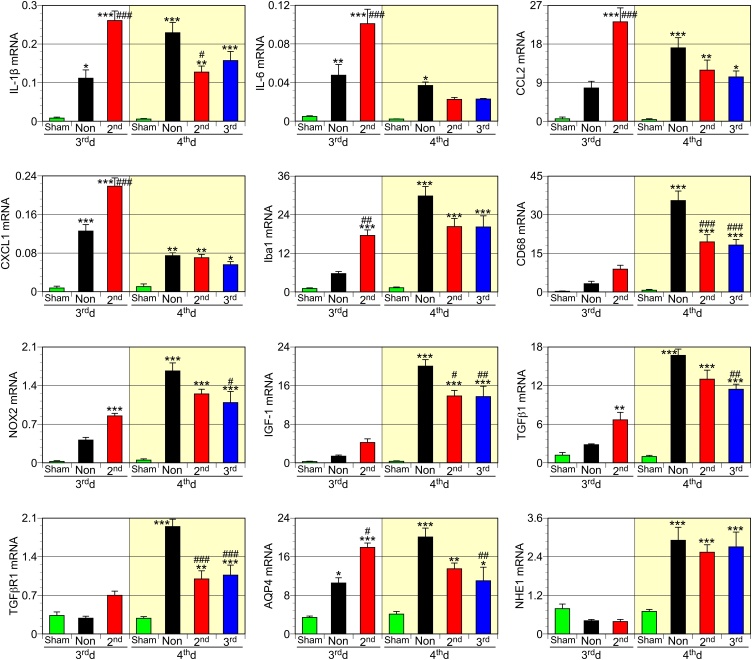
Effects of TBI and exercise on mRNA expression in injured brain tissues dissected on the 3^rd^ d or 4^th^ d. The 2^nd^dEX increased the expression of mRNA encoding IL-1β, IL-6, CCL2, CXCL1, and Iba1 on the 3^rd^ d. mRNA for Iba1, CD68, NOX2, IGF-1, TGFβ1, TGFβR1, AQP4, and NHE1 was increased in NonEX rat tissues on the 4^th^ d, whereas the expression levels of IL-1β, IL-6, and CCL2 mRNA decreased or tended to decline. Exercise decreased mRNA expression for IL-1β, CD68, NOX2, TGFβ1, and TGFβR1 on the 4^th^ d. Data (n = 4) are shown as mean ± SEM. * # $, ** ## $$, *** ### $$$ indicate P < 0.05, 0.01, 0.001, respectively. * vs. NonEX on the 3^rd^ d; # vs. 2^nd^dEX on the 3^rd^ d; $ vs. NonEX on the 4^th^ d.

To elucidate the mechanisms underlying the aggravating effects of the one-time exercise session during the early-onset period, cDNA was prepared from the dissected injured brain tissue for qPCR analysis (Fig. 5). Although physical exercise is known for its anti-inflammatory effects (Piao et al., 2013; Zhang et al., 2016), qPCR using cDNA from the tissues dissected on the 3^rd^ d showed that 2^nd^dEX rats showed an increased expression of mRNA encoding proinflammatory cytokines IL-1β and IL-6. Similarly, the mRNA expression of chemokines CCL2 and CXCL1 were increased in response to the 2^nd^ dEX on the 3^rd^ d. These chemokines are known to enhance infiltration of monocytes (Abe et al., 2018; Tei et al., 2013) and granulocytes (Szmydynger-Chodobska et al., 2009). Yet, the exercise-induced proinflammatory reaction disappeared on the 4^th^ d. The increase in Iba1 and CD68 mRNA levels in the NonEX group on the 4^th^ d may be correlated with the accumulation of activated microglia and blood-borne macrophages. In contrast, 2^nd^dEX and 3^rd^dEX rats showed reduced expression of these microglia/macrophage markers.

NADPH-oxidase 2 (NOX2) is mainly expressed by macrophages in TBI pathology and responsible for reactive oxygen species (ROS) generation (Abe et al., 2018; Li et al., 2017a). NOX2 mRNA expression was increased in the NonEX brain, and the 3^rd^d exercise suppressed the expression on the 4^th^ d. Expression of IGF-1, a presumably ameliorating factor in brain injury (Kazanis et al., 2004; Mangiola et al., 2015), was increased from the 4^th^ d probably because of the accumulation of macrophages (Smirkin et al., 2010). The expression of the anti-inflammatory cytokine transforming growth factor (TGF) β1 and its receptor TGFβR1 was increased in the TBI brain on the 4^th^ d. Both the 2^nd^dEX and 3^rd^dEX rats showed suppressed TGFβ1 and TGFβR1 expression on the 4^th^ d. Aquaporin 4 (AQP4) and Na^+^/H^+^ exchanger 1 (NHE1) are responsible for the induction of brain edema after TBI (Nishioka et al., 2016). The 2^nd^ d exercise slightly increased AQP4 on the 3^rd^ d, but both the 2^nd^dEX and 3^rd^dEX rats showed slight suppression of AQP4 expression on the 4^th^ d. NHE1 expression was not affected by exercise.

### 3.4 Changes in protein expression and phosphorylation after TBI and exercise

**Fig. 6:**
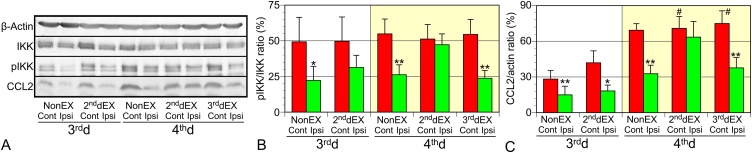
Analyses of protein contents and protein phosphorylation after TBI and exercise. (A) Representative immunoblots on β-actin, IKK, pIKK, and CCL2 expressed in non-injured contralateral (Cont) and injured ipsilateral (Ipsi) brain tissue. (B) Comparison of pIKK-immunoreactive bands normalized to IKK bands. pIKK-immunoreactivity was reduced in the Ipsi tissue, except for the samples from the 2^nd^dEX on the 4^th^ d. (C) CCL2 protein expression was decreased in the Ipsi tissue, except for the samples from the 2^nd^dEX on the 4^th^ d. CCL2 was also increased in the EX groups on the 4^th^ d, compared with the NonEX group on the 3^rd^d (#, P < 0.05 vs. cont of NonEX on the 3^rd^d; ANOVA and Tukey’s post hoc test). Data (n = 5) are shown as mean ± SD. *, ** indicates P < 0.05, 0.01, respectively: compared with the expression in the Cont tissues using paired two-tailed *t*-test.

Nuclear factor κB (NFκB) is a critical transcription factor for proinflammatory reactions, such as the expression of IL-1β and IL-6 (Ishii et al., 2015). NFκB translocates into the nuclei of cells after IκB kinase (IKK) is phosphorylated. Therefore, the effects of exercise on the phosphorylation of IKK were investigated (Fig. 6). Phosphorylated IKK (pIKK)-immunoreactivity was reduced in tissue resected ipsilaterally, whereas only those from the 2^nd^dEX rats on the 4^th^ d contained almost comparable amounts of pIKK in contralateral tissue. CCL2 protein content was also reduced in the ipsilateral tissues except for the one obtained from the 2^nd^dEX rats on the 4^th^ d.

### 3.5 Changes in blood-borne macrophages and microglia

**Fig. 7:**
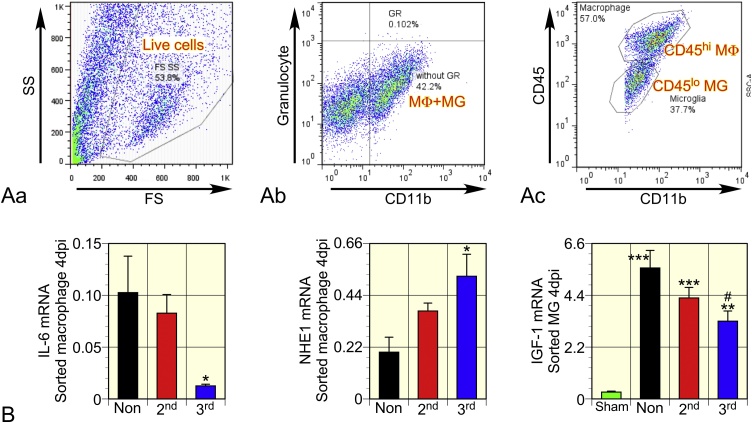
Resident microglia and blood-borne macrophages were analyzed separately by flow cytometry (A), and mRNA expression by sorted cells was evaluated (B). (A) Macrophages and microglia were identified in the total live cell fraction (Aa) and were separated using anti-granulocyte and anti-CD11b antibodies (Ab). Macrophages and microglia were separated based on the immunoreactivity to CD45; macrophages belonged to CD45^hi^ population and microglia to CD45^lo^ one (Ac). These are the representative results obtained from a NonEX rat brain tissue. (B) cDNA was prepared on the 4^th^ d from sorted macrophages and microglia from sham (n = 4), NonEX (n = 4), 2^nd^dEX (n = 5), and 3^rd^dEX (n = 5) rats. Enough number of macrophages could not be obtained from the sham rats for qPCR. mRNA expression of IL-6 by macrophages and that of IGF-1 by microglia from the 3^rd^dEX rats was suppressed, and NHE1 mRNA expression by macrophages was increased. Data are shown as mean ± SEM. * indicates P < 0.05, * vs. NonEX.

Blood-borne macrophages and resident microglia express various factors that presumably affect the outcome of TBI. The cells were sorted separately on the 4^th^ d based on CD45 expression level, cell size, and cell granularity (Fig. 7A), as described previously (Abe et al., 2018). cDNA was prepared from macrophages and microglia. The effects of exercise on expression of mRNA encoding iNOS, IL-1β, IL-6, IGF-1, TGFβ1, and NHE1 were investigated. Consequently, macrophages and microglia from the 3^rd^dEX rats showed suppressed IL-6 and IGF-1 expression, respectively (Fig. 7B). Macrophages from the 3^rd^dEX rat increased NHE1 expression. Cells from the 2^nd^dEx rats showed weaker expressional changes on the 4^th^ d.

## 4 Discussion

The present one-time exercise model involved a forced, slow, 10-min walk on a treadmill, and it did not cause either the elevation of plasma corticosterone levels or changes in adrenal gland weight, although the continuous forced exercise increases corticosterone levels in circulation (Hayes et al., 2008; Nishioka et al., 2016) or in feces (Svensson et al., 2016). It has not been settled whether the effects of increased corticosterone level caused by forced exercise are beneficial or not. The one-time exercise session did not affect the body weight gain after TBI. Taken together, the present one-time exercise session was not considered to be very stressful for TBI rats. Yet, the exercise, particularly that on the 2^nd^ d, aggravated the TBI-caused motor deficits as revealed by cylinder and beam tests two or three weeks later. The 2^nd^ d exercise tended to increase the TBI-induced hyperactivity in the open-field test that might be related to psychological problems (Forrest et al., 2014). This exercise-induced aggravation was not associated with an increase in brain tissue loss. Collectively, even if it is a low-intensity exercise and not very stressful, initiation of exercise too early can become a deleterious intervention for patients with brain injuries. These results are in accordance with those obtained in a randomized control trial targeting ischemic stroke patients, in which mobilization (including sitting, standing, and walking) initiated within 24 h after onset did not result in an improved outcome (Tong et al., 2019). Similar randomized controlled trials demonstrated that the higher dose, very early mobilization is associated with a reduction in the odds of a favorable outcome at 3 months (group, 2015; Winstein et al., 2016). These clinical studies seem to overcome the earlier preclinical and clinical reports describing the beneficial effects of early initiation of exercise within 24 h (Bernhardt et al., 2008; Tian et al., 2013; Zhang et al., 2012).

The most probable cause for the deleterious effects of exercise initiated early may be the induction of proinflammatory reactions, as has been suggested (Li et al., 2017b). In group 2^nd^dEX, increased expression of proinflammatory cytokines and chemokines was observed on the following day, whereas the increase was not observed in group 3^rd^dEX on the day following the intervention. CCL2 has been suspected of its deleterious effects on rat TBI model (Abe et al., 2018; Gyoneva and Ransohoff, 2015) because the chemokine recruits proinflammatory, ROS-generating macrophages. Increased CCL2 expression was observed in group 2^nd^dEX compared to the NonEx but not the 3^rd^dEX. Furthermore, expression of TGFβ1 and its receptor was inhibited in both the 2^nd^ and 3^rd^dEX groups to a similar extent on the 4^th^ d. Expression of IGF-1, which may be involved in the restoration of injured tissue (Kazanis et al., 2004; Mangiola et al., 2015; Smirkin et al., 2010), was slightly suppressed.

Conversely, exercise during the early period may also have favorable effects. As has been described that early exercise initiated at 24 h after onset of ischemic brain injury for 3 days ameliorates brain edema (Nishioka et al., 2016), the present one-time exercise showed a tendency to suppress the expression of AQP4, a responsible factor for brain edema. A marker for phagocytes, CD68 is expressed by macrophages and microglia in the injured brain tissue (Aono et al., 2017; Sugimoto et al., 2014). Phagocytosis by these cells is reported to enhance neuronal loss by eliminating cells that are still viable (Neher et al., 2013). The present exercise suppressed CD68 expression on the 4^th^ d, which may lead to the amelioration of TBI. Group 3^rd^dEX showed a suppressed expression of NOX2, which generates ROS in the injured brain (Abe et al., 2018). This may also be another ameliorating change caused by exercise in the early period.

Thus, the exercise at 24 h or 48 h may exert not only deleterious but also ameliorative effects on the injured brain. However, in our experiment in a rat TBI model, exercise aggravated TBI-induced motor impairments. Therefore, the overall effects of exercise too early after TBI onset may be deleterious. Forced exercise elevates corticosterone levels, leading to the amelioration of severe ischemic brain injury (Hayes et al., 2008; Nishioka et al., 2016). However, the present one-time low-intensity exercise did not increase the corticosterone level, suggesting that the overall deleterious effects may not be attributable to the changes in the hypothalamic–pituitary–adrenal axis. Identification of molecules or factors related to the deleterious effects of exercise at the early time points should make the exercise more safely applicable for many patients with severe brain injuries.

In this sense, IKK might be a critical molecule. NFκB is a transcription factor profoundly responsible for proinflammatory reactions such as the expression of IL-1β and IL-6. NFκB translocates into the cellular nuclei after IκB is phosphorylated by phosphorylated IKK (Islam et al., 2018). The ratio of pIKK compared with total IKK was reduced in the injured ipsilateral hemisphere, suggesting the presence of suppressive factors for proinflammatory reactions in the injured brain. TGFβ1 prevents the phosphorylation of IKK in a sustained manner (Islam et al., 2018); thus, it could be a suitable target for IKK inhibition. The TGFβ1-mediated signal seems to be inhibited by the early initiation of exercise as revealed by qPCR showing a lessened expression of TGFβ1 and TGFβR1. Reportedly, CCL2 is deleterious for injured brains (Abe et al., 2018; Tei et al., 2013), and its expression is positively driven by NFκB (Giraud et al., 2010). Thus, the deleterious effects of exercise during an early period may be at least partly attributable to enhanced phosphorylation of IKK and resulting in increased expression of CCL2. Therefore, various types of anti-inflammatory agents that inhibit IKK activity (Islam et al., 2018) and/or CCL2 expression (Gyoneva and Ransohoff, 2015) may have the potential to prevent the unfavorable effects of the early initiation of exercise after TBI.

In conclusion, the early initiation of exercise may cause proinflammatory responses that are potentially harmful for injured brains, and therefore, it should be paid more attention for the initiation timing. The present study suggest that rehabilitation exercise programs should be initiated after 48 h of TBI onset. However, even when the exercise is initiated one week after the onset of murine TBI prepared by cortical impact, increased IL-1β and complement C1qb expression has still been observed in the chronic phase (5 weeks after injury) compared to late exercise initiation at 5 weeks (Piao et al., 2013). Therefore, the timing of the initiation should be determined taken the severity and the nature of the brain injuries for the better outcome.

## Author contributions

S. Taguchi and J. Tanaka designed the study. S. Taguchi did animal experiments. K. Miyanishi advised on behavioral studies. M.E. Choudhury, N. Abe and K. Kameda did flow cytometry experiments and some of in vitro experiments. Y. Nakanishi did qPCR experiments. H. Yano and T. Yorozuya analyzed the data and advised the experimental design. J. Tanaka wrote the manuscript. All authors approved the final version of the manuscript.

## Conflict of interest

The authors have nothing to declare.
