# Supplementary material for: Aggravating effects of treadmill exercises during the early-onset period in a rat traumatic brain injury model: When should rehabilitation exercises be initiated?
Source: IBRO Rep. 2019 Oct 22;7:82–9. doi: 10.1016/j.ibror.2019.10.002 (PMC6838542; doi:10.1016/j.ibror.2019.10.002)
Supplement: Supplementary file 1 [file mmc1.docx]

Supporting file for IBRO Reports Volume 7, December 2019, Pages 82-89

**Aggravating effects of treadmill exercises during the early-onset period in a rat traumatic brain injury model: When should rehabilitation exercises be initiated?**

Satoru Taguchi^1^, Mohammed E. Choudhury^1^, Kazuya Miyanishi^1^, Yuiko Nakanishi^1^, Kenji Kameda^2^, Naoki Abe^3^, Hajime Yano^1^, Toshihiro Yorozuya^3^, Junya Tanaka^1^

^1^Department of Molecular and Cellular Physiology, Graduate School of Medicine, Ehime University, Toon, Ehime, Japan

^2^Advanced Research Support Center, Division of Analytical Bio-Medicine, Ehime University, Toon, Ehime, Japan

^3^Department of Anesthesia and Perioperative Medicine, Graduate School of Medicine, Ehime University, Toon, Ehime, Japan

Supplementary Tables

| Supplementary Table 1 PCR Primers | |
| --- | --- |
| Primers | Sense/anti-sense |
| AQP4 | GAATCCAGCTCGATCCTTTG |
|  | CTCCATGTAGCTCCCTTTCG |
| CCL2 | TTGTCACCAAGCTCAAGAGA |
|  | CACATTCAAAGGTGCTGAAG |
| CD68 | AATGTGTCCTTCCCACAAGC |
|  | GAGGCAGCAAGAGAGATTGG |
| CXCL1 | AGACAGTGGCAGGGATTCAC |
|  | ACTTGGGGACACCCTTTAGC |
| GAPDH | GAGACAGCCGCATCTTCTTG |
|  | TGACTGTGCCGTTGAACTTG |
| Iba1 | GTCCTTGAAGCGAATGCTGG |
|  | CATTCTCAAGATGGCAGATC |
| IGF1 | GGCATTGTGGATGAGTGTTG |
|  | CTTCTGAGTCTTGGGCATGT |
| IL1β | CACCTTCTTTTCCTTCATCTTTG |
|  | GTCGTTGCTTGTCTCTCCTTGTA |
| IL6 | CTGATGTTGTTGACAGCCAC |
|  | CAGAATTGCCATTGCACAAC |
| iNOS | AGGGAGTGTTGTTCCAGGTG |
|  | TCCTCAACCTGCTCCTCACT |
| NHE1 | CAACGGCTGCGGTCCTATAAC |
|  | GGTGAAGACATCATCGGTGC |
| NOX２ | GCTGGGATTGGAGTCACG |
|  | GCACAGCCAGTAGAAGTAGATCTTT |
| TGFβ1 | TGAGTGGCTGTCTTTTGACG |
|  | GGTTCATGTCATGGATGGTG |
| TGFβR1 | AACGTTCATGGTTCCGAGAG |
|  | ATGTGAAGATGGGCAAGACC |

| Supplementary Table 2 Antibodies for flow cytometry | | | |
| --- | --- | --- | --- |
| Antigen | Antibody | Clone | Source |
| CD11b/c APC | Human monoclonal | REA325 | Miltenyi Biotec (Bergisch Gladbach, Germany) |
| CD45 PE | Human monoclonal | REA504 | Miltenyi Biotec |
| Granulocytes FITC | Human monoclonal | REA535 | Miltenyi Biotec |

| Supplementary Table 3 Antibodies for Immunoblotting | | | |
| --- | --- | --- | --- |
| Antigen | Antibody | Clone | Source |
| β-actin | Mouse monoclonal | AC-15 | Sigma-Aldrich |
| IKK-α | Rabbit polyclonal |  | Cell Signaling Technology, Danvers, MA |
| P-IKK-α/β | Rabbit monoclonal | 16A6 | Cell Signaling Technology |
| MCP-1 | Goat polyclonal |  | Santa Cruz Biotechnology, Dallas, TX |
